# Clinical observation of male bulbar urethral strictures resulting from straddle injuries associated with falling from heights and riding activities

**DOI:** 10.1186/s12893-024-02435-x

**Published:** 2024-05-11

**Authors:** Wei Li, Libo Man, Guanglin Haung

**Affiliations:** Department of Urological Surgery, Beijing Jishuitan Hospital, Capital Medical University, Beijing, 100096 China

**Keywords:** Bulbar urethra, Stricture or occlusion, Straddle injury, Falling from height, Riding activity

## Abstract

**Objectives:**

To retrospectively investigate and analyze the characteristics of male bulbar urethral strictures or occlusions resulting from straddle injuries caused by falling from heights and riding activities.

**Methods:**

The study included 56 patients with a history of straddle injury, who were divided into two groups: the falling group (*n* = 29) and the riding group (*n* = 27). All patients underwent urethroscopy and X-ray urethrography, followed by urethrotomy and anastomotic procedure. Both urethral and suprapubic catheters were retained for one month postoperatively. Subsequent follow-up assessments were conducted within one month to one year after surgery.

**Results:**

The clinical data of two groups were analyzed. The average ages were 40.1 ± 11.2 (falling group, aged 18–59) and 26.8 ± 4.4 (riding group, aged 19–35), *P* < 0.05. In the falling group, 21 cases (72.4%) had offspring, while in the riding group, only 3 cases (11.1%) had offspring, *P* < 0.05. The stricture segments in the falling group were predominantly located in the proximal part of the bulbar region (89.7%), whereas in the riding group they mainly found in the distal part (96.3%), *P* < 0.05. In terms of urethrography results, the average lengths of stricture segments were measured as 17.6 ± 2.8 mm and 15.5 ± 4.6 mm respectively, *P* < 0.05. During surgery, the average lengths of stricture segments were recorded as 19.0 ± 2.5 mm and 17.4 ± 6.1 mm, *P* > 0.05. In the falling group, 20 cases (69.0%) involved bulbocavernosus muscle injury, *P* < 0.05. In the riding group, 5 cases (18.5%) involved corpus cavernosum injury, *P* < 0.05. After one month of the operation, all cases were able to pass through the 16Fr urethroscope without any apparent urethral strictures or complications observed in urethrography results. The maximum urinary flow rate for all cases exceeded 15 ml/s. Two months and one year after the operation, all cases experienced smooth urinary flow and ejaculation without any disorders reported. 3 cases (10.3%) in the falling group and 7 cases (25.9%) in the riding group complained of urethral stretching pain during erection, *P* > 0.05.

**Conclusions:**

Male bulbar urethral strictures or occlusions resulting from straddle injuries associated with falling from heights and riding activities exhibit distinct characteristics, necessitating the development of a comprehensive surgical plan tailored to the specific features of each condition and the diverse age groups affected.

**Supplementary Information:**

The online version contains supplementary material available at 10.1186/s12893-024-02435-x.

## Background

Straddle injury is a common cause of blunt bulbar urethral injuries in men, often resulting in urethral stricture or occlusion after the injury [1]. The injury mechanism occurs when the perineum impacts a hard object, compressing the bulb urethra against the pubic symphysis and resulting in rupture of the urethra at the site of compression [1, 2]. Falling from a height and riding activity accident are two common causes of straddle injuries in clinical, resulting in different characteristics of urethral strictures or occlusions [1–3]. The following retrospective observational analysis was conducted in our department on patients with urethral stricture caused by straddle injuries, and the findings are reported as follows.

## Patients and methods

### Patients

The study was approved by the Ethics Committee, No.20,211,212. The clinical data of male patients with urethral stricture caused by staddle injury, who were admitted to the Department of Urology at Beijing Jishuitan Hospital from January to December 2022, were collected. Inclusion criteria: (1) patients with a clear history of saddle injury, where the cause of injury was falling from a height and hitting a hard object in the perineum or having a collision while riding a motorcycle, bicycle, go-karting, or other means of transportation; (2) not having undergone urethroplasty or anastomosis; (3) more than three months after the injury; (4) with a suprapubic catheter; (5) urethroscopy or urethrography showing urethral stricture or occlusion requiring further surgical treatment. Exclusion criteria: (1) combined with an open perineal skin injury; (2) combined with other injuries, such as pelvic fracture, testicular or epididymal injury, bladder rupture, etc.; (3) with contraindications to urethral anastomosis, reconstructive surgery, or anesthesia; and (4) acute urinary tract infection. A total of 56 patients were enrolled and divided into two groups according to the cause of injury: 29 cases in the falling group and 27 cases in the riding group.

### Methods

The enrolled patients in both groups underwent routine medical history collection, physical examination, and other necessary preoperative preparations. Urethroscopy and X-ray urethrography were used to determine the location, length, and degree of stricture, as well as the presence of other complications such as urethral diverticulum, fistula, or sinus.

#### Urethroscopy

After local anesthesia, a flexible electronic cystoscope (16 F, Olympus, Japan) was used to enter the urethra through the external and internal orifices until it could no longer pass. Both ends of the urethral stricture segment were examined.

#### X-ray urethrography

X-ray machine (Sonialvision Safire II/4124A3326005, SHIMADZU, Japan). The contrast medium used was Iodixanol-based (160 mg I/ml, General Electric, USA), which was diluted 1:1 with normal saline. To protect other organs during X-ray urethrography, the patient wore a lead coat, hat, neck brace, etc. Voiding cystourethrography and retrograde urethrography were performed following the methods mentioned in the literature [4], including injecting contrast material through the suprapubic catheter and external urethral orifice respectively. The body positions included supine position as well as right oblique positions of 30° and 60°. After filling the distal and proximal ends of the stricture segment with contrast medium, radiography was performed to visualize and observe the ends and surrounding conditions of the stricture segment. Post-processing and length measurement were done using Carestream PACS 11.0 software.

#### Surgical methods and perioperative management

Urethrotomy surgery was performed on all patients in both groups. The median incision approach was selected from the scrotum to the perineum, based on the preoperative examination findings determining the location of the stricture segment [5]. The normal urethra at both ends of the stricture segment was dissected, followed by excision of the stricture segment with recording its length. In cases where the stricture segment was excessively long, incising the septum of corpus cavernosum was performed to reduce tension during anastomosis. The normal urethra should be sutured at both ends using the 4 − 0 absorbable sutures.

Postoperatively, both urethral and suprapubic catheters were retained for a duration of 1 month, after which patients voided spontaneously upon catheter removal. Immediate postoperative evaluations included urethroscopy, urethrography, and measurement of maximum urinary flow rate.

Follow-up assessing voiding function, the lower urinary tract symptoms (LUTS), erectile function (EF), and ejaculatory function (EJF) were conducted 2-month and 1-year after surgery, using Patient Reported Outcome Measurements (PROMs).

### Evaluation indicators

The results of urethroscopy and urethrography were used to compare the location, length, degree of stricture, and the presence of other complications. The actual length of the stricture segment, operation time, blood loss, whether to incise the corpus cavernosum septum, etc., and the situation of scar involving the surrounding organs (corpus cavernosum/bulbocavernosum muscle) were recorded during the operation. 1-month after surgery, urethroscopy and urethrography were used to observe the urethral patency, anastomotic healing, whether there were complications around the urethra, maximum urinary flow rate.

The LUTS were evaluated using the International Prostate Symptom Score (IPSS). The IPSS was categorized as follows: asymptomatic (0), mild symptoms (1–7), moderate symptoms (8–19), and severe symptoms (20–35). The Quality of Life (QoL) score ranged from 0 to 6, with 0 indicating the best. EF was assessed using the International Index of Erectile Function (IIEF-5). The EF was classified into different levels: no erectile dysfunction(ED) (22–25), mild ED (17–21), mild-to-moderate ED (12–16), moderate ED (8–11), and severe ED (1–7). EJF was evaluated using the Ejaculatory Domain-Male Sexual Health Inventory Questionnaire (Ej-MSHQ). A total Ej-MSHQ score between 28 and 35 indicated good ejaculatory function, a score between 22 and 27 indicated average ejaculatory function, while a score below 22 indicated ejaculatory dysfunction.

### Statistical methods

SPSS software version 19.0 was used for statistical analysis. The measurement data were expressed as mean ± standard deviation $\left( {\vec x \pm s} \right)$. Two independent sample *t* test was performed if it was normal distribution, and rank sum test was performed if it was not. *P* < 0.05 was considered statistically significant. Chi-square test or Fisher’s exact test was used to compare the count data, and *P* < 0.05 was considered statistically significant.

## Results

The clinical data of 56 cases were analyzed and presented in Table 1.

**Table 1:** Comparison of preoperative and intraoperative conditions between the two groups

	Total	Falling group	Riding group	*P*-value
Number of cases n (%)	56	29 (51.8)	27 (48.2)	
Mean age & range (years)	33.7 ± 10.8 (18–59)	40.1 ± 11.2 (18–59)	26.8 ± 4.4 (19–35)	< 0.05
Have offspring n (%)				
Yes	24 (42.9)	21 (72.4)	3 (11.1)	< 0.05
No	32 (57.1)	8 (27.6)	24 (88.9)	
Location of stricture segment n (%)				
Proximal part of the bulbar	27 (48.2)	26 (89.7)	1 (3.7)	< 0.05
Distal part of the bulbar	29 (51.8)	3 (10.3)	26 (96.3)	
Degree of stenosis n (%)				
Stricture	14 (25.0)	7 (24.1)	7 (25.9)	> 0.05
Occlusion	42 (75.0)	22 (75.9)	20 (74.1)	
Stricture segment length (mm)				
Urethrography	16.6 ± 3.9	17.6 ± 2.8	15.5 ± 4.6	< 0.05
Operation	18.2 ± 4.6	19.0 ± 2.5	17.4 ± 6.1	> 0.05
Operation duration (min)	97.8 ± 11.7	93.6 ± 8.7	102.3 ± 13.0	< 0.05
Amount of blood loss (ml)	40.3 ± 11.0	39.5 ± 8.8	41.1 ± 13.0	> 0.05
Scar area n (%)				
Bulbocavernosus muscle	20 (35.7)	20 (69.0)	0 (0)	< 0.05
Corpus cavernosum	5 (8.9)	0 (0)	5 (18.5)	< 0.05

**Fig. 1:**
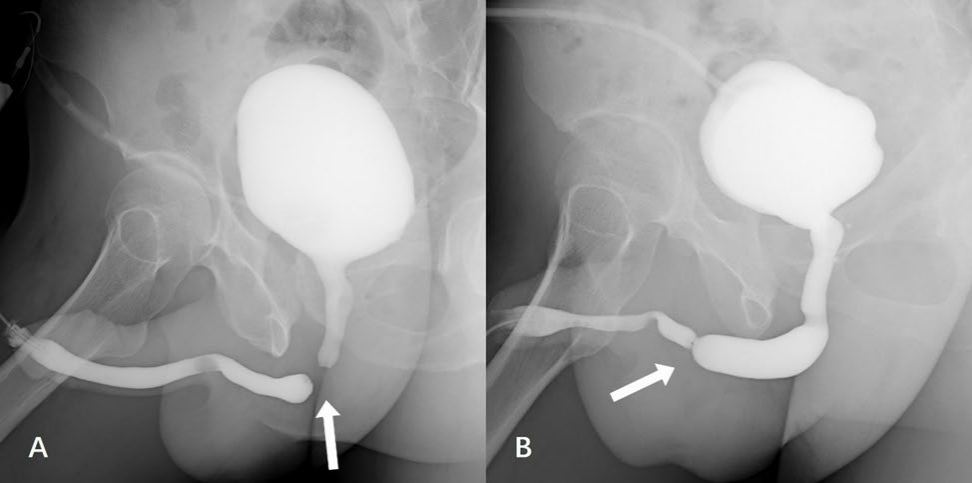
Preoperative urethrography was performed in both groups. (**A**) Urethral occlusion was caused by a straddle injury from falling. The arrow indicates that the occlusion segment is located at the proximal part of the bulbar urethra, and its direction represents the direction of the external force at the time of injury. (**B**) Preoperative urethrography was performed in both groups. Urethral stricture was caused by a straddle injury from riding a motorcycle. The white arrow indicates that the stricture is located at the distal part of the bulbar urethra, and its direction represents the direction of the external force at the time of injury

No complications were found during preoperative urethroscopy and urethrography. The location of stricture segments in urethrography was shown in Fig. 1. All operations were successfully completed. In only one case in the riding group, the septum of the corpus cavernosum was incised, with a stricture segment length of 45 mm. The maximum length of the remaining cases was 25 mm in the falling group and 22 mm in the riding group. The two groups exhibited certain differences, such as the length of stricture segments in urethrography and the duration of operations, which were statistically significant but not clinically significant.

One month after the operation, all cases were able to pass through the 16Fr urethroscope. The anastomosis of all cases healed well without any obvious strictures observed. Urethrography revealed no apparent urethral strictures or complications. The maximum urinary flow rate for all cases exceeded 15 ml/s.

After a 2-month and 1-year follow-up, all cases reported unobstructed urinary flow, as well as smooth ejaculation without any disorders. The PROMs were analyzed and presented in Table 2. During erection, there were 3 cases (10.3%) in the falling group and 7 cases (25.9%) in the riding group that complained of urethral stretching pain; however, this difference was not statistically significant (*P* > 0.05).

**Table 2:** The follow-up assessments at 2-month and 1-year using PROMs, n (%)

Follow-up Period	PROMs	Falling group	Riding group	*P*-value
2-month	**IPSS**			
	asymptomatic, 0	14 (48.3)	16 (59.3)	> 0.05
	mild symptoms, 1–7	15 (51.7)	11 (40.7)	
	**QoL**			
	0	10 (34.5)	12 (44.4)	> 0.05
	1	15 (51.7)	12 (44.4)	
	2	3 (10.3)	3 (11.1)	
	3	1 (3.4)	0 (0)	
	**IIEF-5**			
	no ED, 22–25	28 (96.6)	25 (92.6)	> 0.05
	mild ED, 17–21	1 (3.4)	2 (7.4)	
	**Ej-MSHQ**			
	good EJF, 28–35	29 (100)	27 (100)	> 0.05
1-year	**IPSS**			
	asymptomatic, 0	20 (69.0)	21 (77.8)	> 0.05
	mild symptoms, 1–7	9 (31.0)	6 (22.2)	
	**QoL**			
	0	19 (65.5)	23 (85.2)	> 0.05
	1	8 (27.6)	3 (11.1)	
	2	2 (6.9)	1 (3.7)	
	**IIEF-5**			
	no ED, 22–25	28 (96.6)	24 (88.9)	> 0.05
	mild ED, 17–21	1 (3.4)	3 (11.1)	
	**Ej-MSHQ**			
	good EJF, 28–35	29 (100)	27 (100)	> 0.05

## Discussion

The male bulbar urethra is commonly affected by straddle injuries to the anterior urethra. In cases of straddle injuries, the bulbar urethra becomes compressed against the pubic symphysis, resulting in a rupture at the site of compression. As recommended by the World Health Organization (WHO) consultation, the bulbar urethra extends from the distal membranous urethra to the proximal penile urethra. It is surrounded by the corpus spongiosum and covered by the bulbospongiosus muscle [1].

The straddle injury occurs in daily life, including falling and riding activities [6], as well as some working occasions that we have observed. It usually involves a section from the penoscrotal junction to the bulbomembranous junction of the anterior urethra [7]. The location of the injury depends on the angle at which the patient strikes the astride structure [8].

When falling from a height, the focus of injury is located at the lowest point of the perineum, which is inferior to the pubic symphysis. Consequently, the injury predominantly affects the proximal segment of the bulbar urethra, including the thicker corpus spongiosum and bulbospongiosus muscle. Following tissue healing and scar formation, this subsequently impairs muscle function and results in dysfunction of ejaculation and terminal urination [9]. However, in cases where riding is involved, the injury point is located anterior to the pubic symphysis, corresponding to the distal segment of bulbar urethra [10, 11]. The thickness of this urethra’s corpus spongiosum is closer to that of penile urethra and lacks bulbospongiosus muscle [7]. Additionally, it is easier for injuries to involve the corpus cavernosum in these cases leading to surrounding scarring that can affect erectile function or cause pain during erection [12]. The use of ultrasound or magnetic resonance imaging has been suggested by some authors for preoperative assessment of injuries surrounding the urethra [13, 14].

Straddle injury usually only affects a short portion of the urethra [7], with 85% of trauma strictures being less than 4 cm in length [15]. The difference in the length of the stricture segment between the two groups did not affect the decision on surgical method in clinical practice. However, there were also cases in the riding group where a longer stenosis segment, up to 45 mm in length, was observed. According to the mechanism of straddle injury, a longer stricture segment indicates prolonged compression on astride structures. We believe this is due to the use of a soft seat that disperses pressure and protects the perineum. When sufficient force is applied, dispersed pressure can also cause injury. As some authors have observed, the reported incidence of anterior urethral injuries was lower [16] and the true incidence of anterior urethral injuries might be far higher than that reported [8].

In order to improve the success rate of anastomosis, it is necessary to remove adequate urethral scars and perform a tension-free anastomosis of the normal urethra at both ends [17]. It is important to utilize its extensibility by further freeing the normal urethra at both ends; additionally, splitting the septum of the corpus cavernosum can shorten the distance of anastomosis and reduce tension during anastomosis. However, this may result in some cases experiencing urethral stretching pain during erection. In cases with long defects, a pedicled flap or free graft such as oral mucosa may be required to replace the urethra [18]. The success of the operation is limited by the survival rate of the graft, which also increases the risk of complications related to donor-site [19].

In particular, the patients in the riding group were younger than those in the falling group; most of them were unmarried and had a high need for fertility and sexual life. Therefore, it is necessary to fully consider before surgery, make detailed surgical plans, reconstruct urethral patency as much as possible, and preserve erectile function and reproductive function.

The utilization of PROMs enhanced the comparability of patients’ symptoms and outcomes, leading to improvements [20, 21]. However, due to the unpredictable nature of trauma and different mechanisms of injury, strictures also exhibit different characteristics. This study is a retrospective analysis conducted under limited inclusion and exclusion criteria; there may still be some bias present but it still reflects the differences in characteristics of urethral stricture caused by these two types of injuries.

## Conclusions

Male bulbar urethral strictures or occlusions resulting from straddle injuries associated with falling from heights and riding activities exhibit distinct characteristics, necessitating the development of a comprehensive surgical plan tailored to the specific features of each condition and the diverse age groups affected.

### Electronic supplementary material

Below is the link to the electronic supplementary material.

Supplementary Material 1

## Data Availability

Data is provided within the manuscript or supplementary information files.
